# An Inexpensive, High-Fidelity Resuscitative Hysterotomy (RH) Model With Hemorrhage Capability

**DOI:** 10.7759/cureus.25582

**Published:** 2022-06-01

**Authors:** Kenneth H Palm, Charles Lei, Ryan Walsh, Jeffrey Heimiller, Joseph Sikon

**Affiliations:** 1 Department of Emergency Medicine, Vanderbilt University Medical Center, Nashville, USA

**Keywords:** obstetric emergencies, emergency obstetric procedures, cardiac arrest in pregnancy, partial task trainer, perimortem cesarean section, resuscitative hysterotomy

## Abstract

Resuscitative hysterotomy (RH) is a rare, time-sensitive, invasive procedure that can be frightening for emergency physicians and yet potentially life-saving for fetus and mother. Several low-cost RH task trainers have been described in the literature. We set out to construct a model using improved synthetic materials for the uterine and abdominal wall and to devise hemorrhage capability. The primary aim of this study was to evaluate the model’s perceived usefulness of its features. Secondarily, we wished to assess the confidence of emergency medicine (EM) residents before and after performing a RH using our task trainer in a simulated environment.

We constructed an inexpensive task trainer that can function both as a table-top model (TTM) and be adapted to a high-fidelity simulator. We created the abdominal wall and uterus from polyurethane carpet padding, subcutaneous fat from upholstery foam, fascia from synthetic chamois, and blood vessels from IV tubing and angio-catheters. We utilized the task trainer during our monthly EM residency simulation conference. After completing a simulation of a gravid female in cardiac arrest requiring a RH on a high-fidelity simulator adaptated model (HFSAM), residents repeated the procedure during debriefing on a TTM. Residents then completed anonymous paper surveys in which they rated aspects of the RH model and their procedural confidence on a 10-point Likert scale.

20 EM residents took part in the RH simulation scenario followed by a TTM demonstration. All (100%) residents completed the survey. 11 (55%) of the residents performed a RH on either the HFSAM or the TTM while the others assisted. The residents rated the overall educational value of the training event as very high (mean 9.8 (SD 0.68)). Both the TTM (mean 8.9 (SD 1.15)) and HFSAM (mean 8.7 (SD 1.29)) were similarly rated as highly realistic. Before the simulation session, residents rated their confidence in performing a RH as low (mean 4.0 (SD 2.62)). After the session, they were much more confident in their ability to perform a RH (mean 7.9 (SD 1.48); P<0.001). Most residents rated bleeding as very important to the utility of a RH model (mean 8.6 (SD 1.74)).

We demonstrate an inexpensive but realistic RH task trainer that can be used as a stand-alone model or adapted to a high-fidelity simulator. A single simulation using the TTM and the HFSAM lead to increased resident confidence in their ability to perform a RH.

## Introduction

Caring for pregnant women who have suffered a sudden physiologic decompensation represents core knowledge for emergency medicine (EM) physicians. Emergency cesarean delivery during maternal cardiac arrest is a potentially life-saving obstetric procedure that EM physicians must know how to perform. Once referred to as a “perimortem cesarean section,” there has been a push in recent years to rename the procedure "resuscitative hysterotomy" (RH) [1]. This change in terminology has taken place to emphasize that rapid removal of the infant from the gravid uterus not only may be beneficial for the fetus but also for the mother because maternal resuscitative efforts are often enhanced following delivery even when there is a fetal demise [2].

Maternal cardiac arrest is a high-acuity, low-occurrence (HALO) event, and few EM physicians will ever perform a RH during their careers. The extremely low incidence makes it difficult for EM physicians to develop and maintain proficiency of this critical skill. In addition, the American Heart Association, American College of Obstetricians and Gynecologists, and Eastern Association for the Surgery of Trauma guidelines recommend initiating a RH within four minutes of maternal arrest to optimize both fetal and maternal resuscitation. Delivery of the newborn ideally should be completed within five minutes of cardiac arrest for the best chance of favorable fetal neurologic outcome [3-5]. These guidelines emphasize the time-critical aspect of the procedure.

Performing a RH can be technically and psychologically overwhelming to the inexperienced practitioner. Simulation-based education using task trainers can enhance physician preparation for and performance of HALO procedures such as RH. While there are commercially manufactured RH task trainers, their high material and maintenance costs limit their availability to educators [6]. There are several homemade, low-cost, low-fidelity task trainers described in the literature that are useful for practicing the steps involved in performing a RH [7-9]. We developed two low-cost, high-fidelity RH models in which we attempted to maximize a realistic appearance, replicate the anatomic tissue layers, and incorporate the ability to modulate active hemorrhage during skin, subcutaneous, and uterine incision. We hypothesize that our models will enhance the technical skills of EM physicians and help mentally prepare them for the procedure.

We first developed a stand-alone table-top model (TTM) for RH, then adapted the model to be incorporated into a high-fidelity simulator.

We presented our abstract at the 20th Annual International Meeting on Simulation in Healthcare, January 18-22, 2020, San Diego, USA. 

## Technical report

Construction of the TTM

Table 1 below lists the components required to construct the TTM.

**Table 1:** Table-top model (TTM) materials and associated cost

Items Purchased	Quantity	Price
open-back maternity plastic torso	1	$17.95
sanded plywood 3/4 in x 2 ft x 4 ft	1	$10.36
memory foam carpet padding 1/4 in x 8 ft x 10 ft	1	$26.31
polyurethane upholstery foam 1 in x 2 ft x 3 ft	1	$9.99
Synthetic chamois 3 ft^2^	1	$5.97
zip ties 4-in 100/pack	1 pack	$1.58
duct tape 1.88 in x 10 yd	1 roll	$2.00
clear plastic wrap 200 ft^2^	1 roll	$1.97
industrial-grade hook-and-loop fastener 10 ft	1 box	$12.60
spray paint 12 oz	2 cans	$9.16
white flour 32 oz	1 bag	$0.94
clear plastic produce bags 1 gallon 50/roll	1 roll	$6.99
IV extension tubing 30 in	11	$10.45
cloth and plastic newborn twin dolls	1	$22.94
placenta life-size with umbilical cord	1	$55.99
angiocatheter 18 gauge x 1.88 in	8	$28.72
Total		$223.92

**Figure 1:**
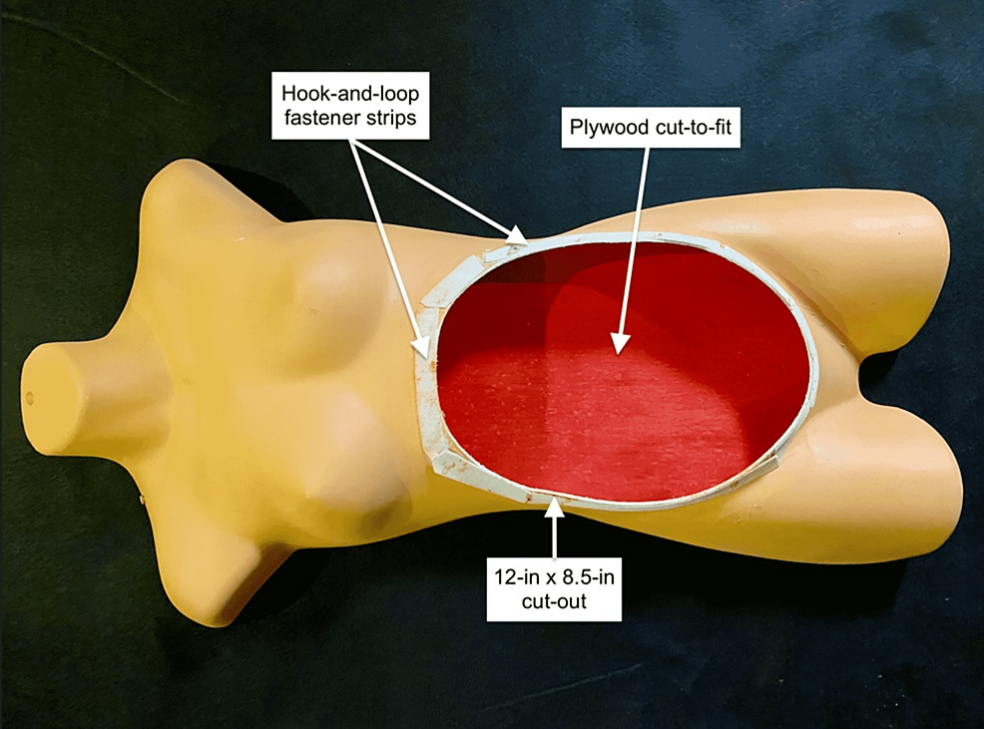
Open-back maternity torso with cut out to accept uterus and abdominal wall layers

A tracing of the perimeter of the plastic torso was made on a piece of sanded plywood. Using a jigsaw, the plywood was cut ⅛ in interior to the tracing to allow it to ﬁt into the back of the torso. A 12-in x 8.5-in oval area of the torso’s abdomen was cut out and the edges sanded lightly (Figure 1).

**Figure 2:**
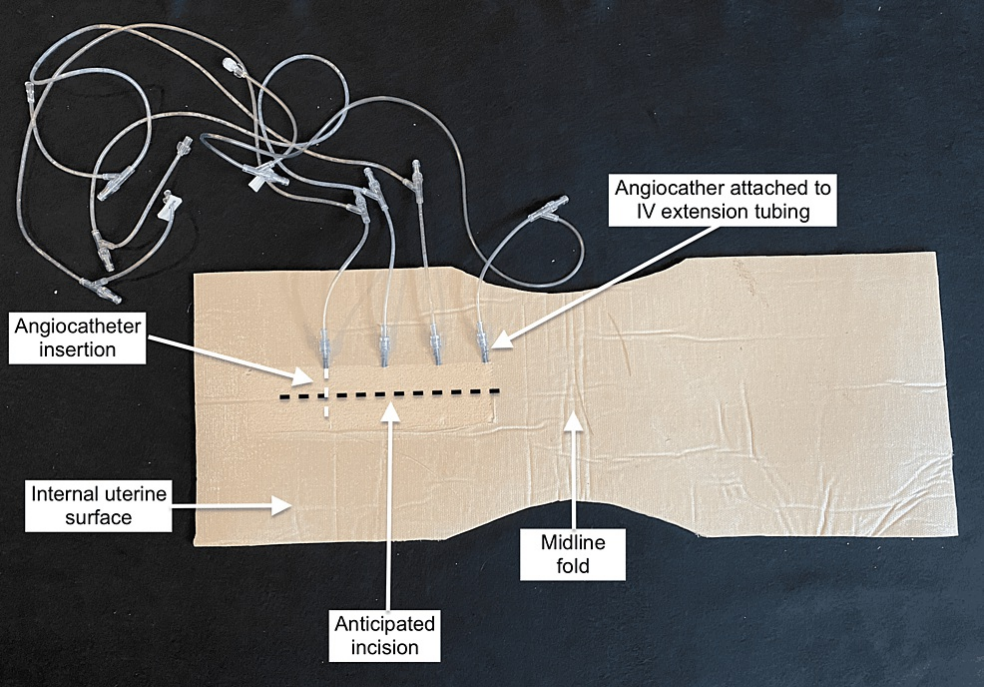
Internal uterine bleeding apparatus

A uterus was formed by cutting a 29-in x 11-in piece of carpet padding and folding it over on itself. Four 20-gauge x 1.88-in angiocatheters were inserted horizontally into the internal uterine wall at the location that a likely uterine incision would be made (Figure 2).

**Figure 3:**
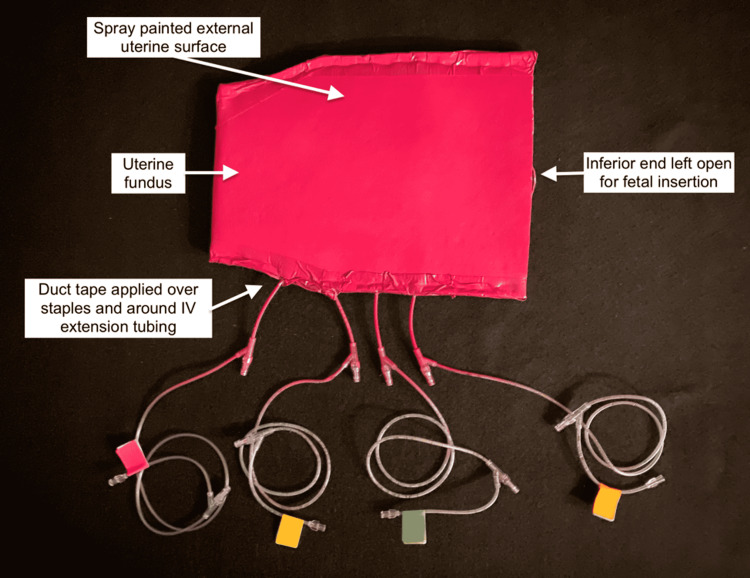
External uterus with secured bleeding apparatus

The catheters were attached to IV extension tubing extended laterally from the uterine edges. The edges of the uterus were stapled together, making sure to avoid the IV extension tubing and leaving the inferior portion open. The stapled edges of the uterus were sealed and reinforced with duct tape. Duct tape was also used to secure the IV extension tubing in place. The uterus was spray-painted a red color (Figure 3).

**Figure 4:**
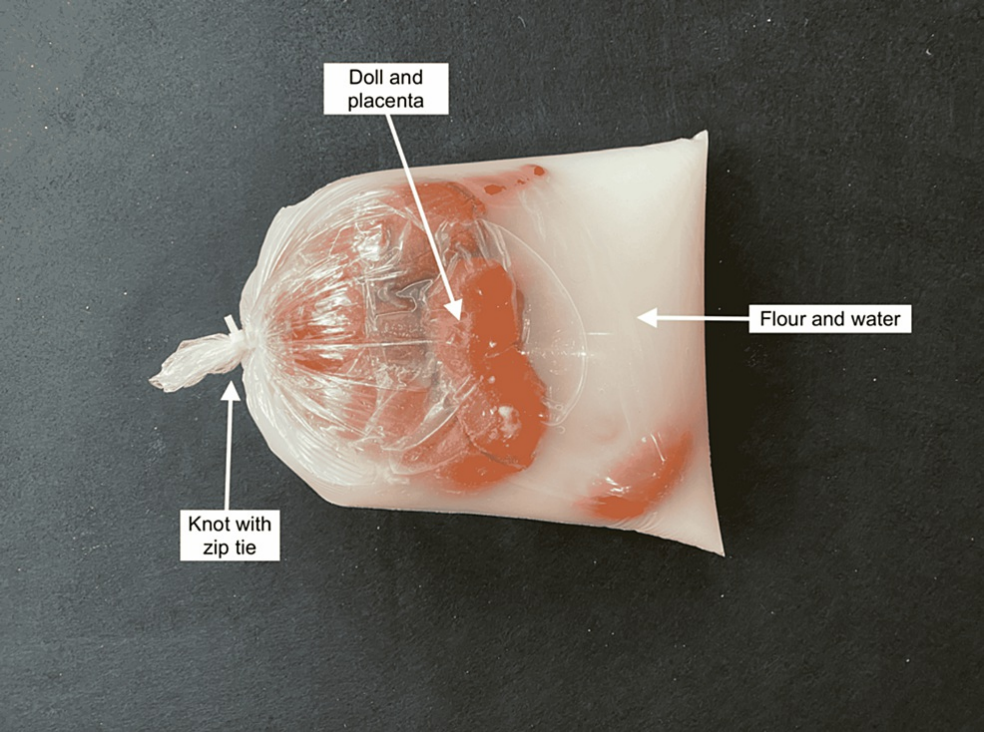
Fetus and placenta in amniotic sac

A cloth-and-plastic newborn doll and placenta were inserted into a clear plastic produce bag. The bag was ﬁlled with water and ½ cup of white ﬂour to simulate amniotic ﬂuid within an amniotic sac. The ends of the plastic bag were tied in a knot and placed into a second plastic bag that was tied and sealed with a zip tie. The bag was shaken lightly to mix the ﬂour and water, giving it a clumpy appearance (Figure 4).

**Figure 5:**
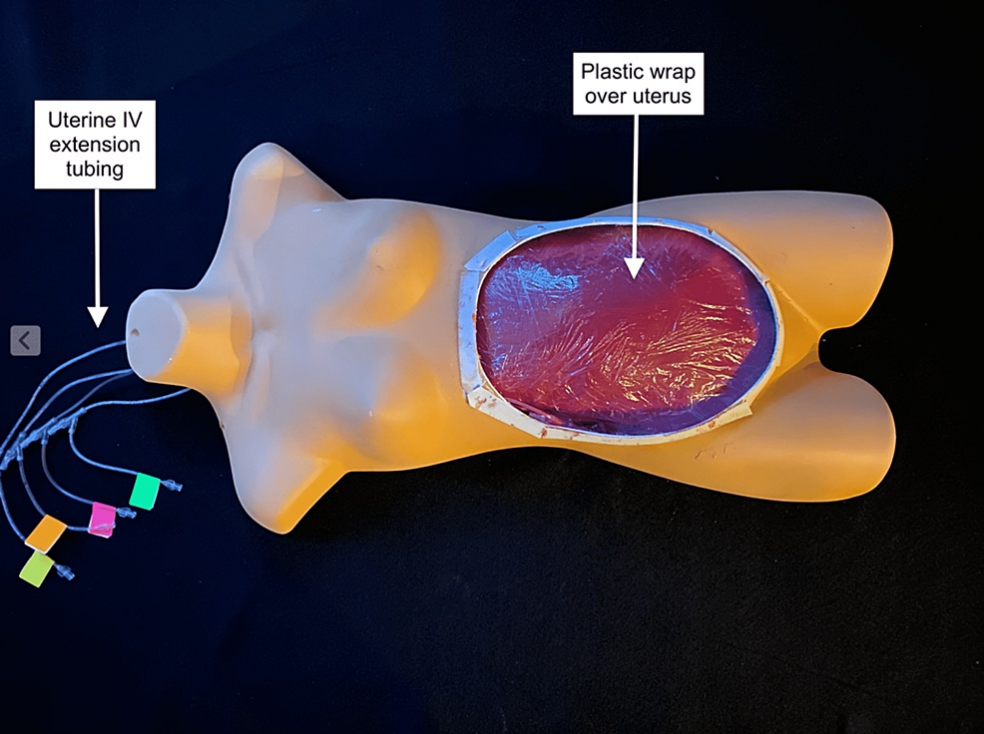
Completed uterus with peritoneal covering

The amniotic sac was inserted into the open inferior end of the uterus and sealed with staples and duct tape. The uterus was wrapped in plastic wrap to simulate the peritoneum (Figure 5).

**Figure 6:**
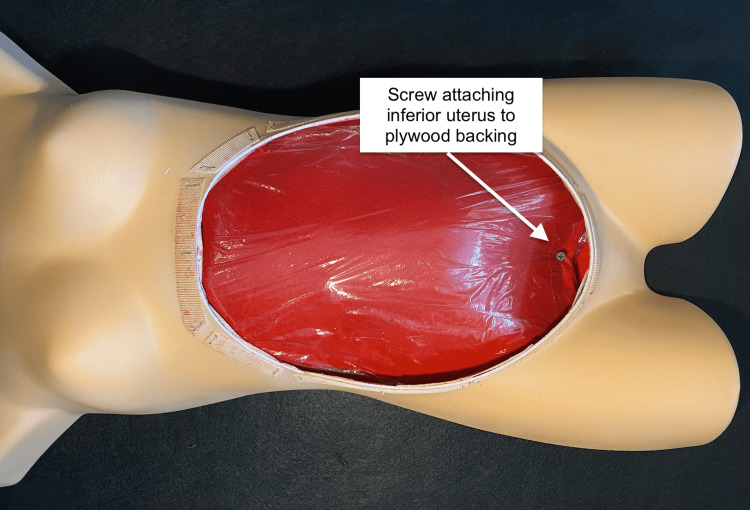
Uterine attachment

A 6-in x 1 ⅝-in drywall screw was used to attach the inferior end of the uterus to the plywood, carefully avoiding penetration of the amniotic sac. Attachment of the uterus to the plywood only at its inferior aspect allowed learners to elevate the uterus out of the abdominal cavity while performing the procedure (Figure 6).

**Figure 7:**
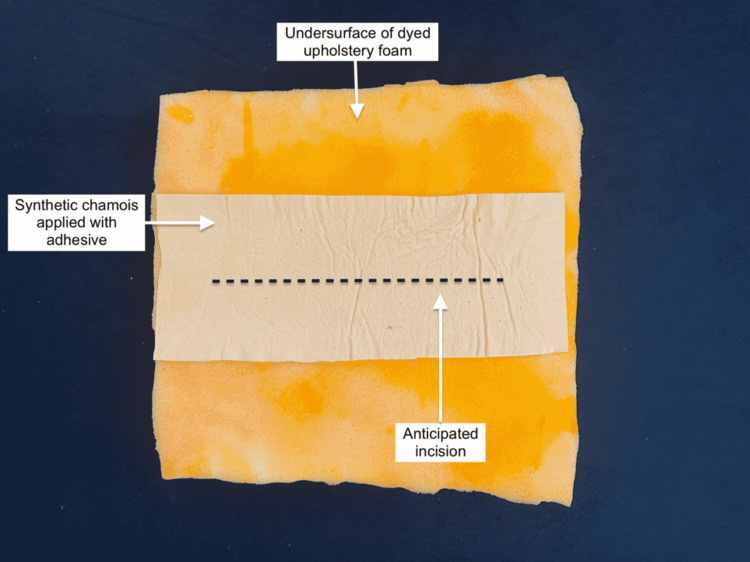
Subcutaneous fat and abdominal fascia

A 1-in x 14-in x 15-in piece of upholstery foam was dyed with yellow food coloring to simulate the appearance of fat. A 6-in x 15-in strip of synthetic chamois was attached vertically to the middle of the foam using spray adhesive to represent the fascial layers (Figure 7).

**Figure 8:**
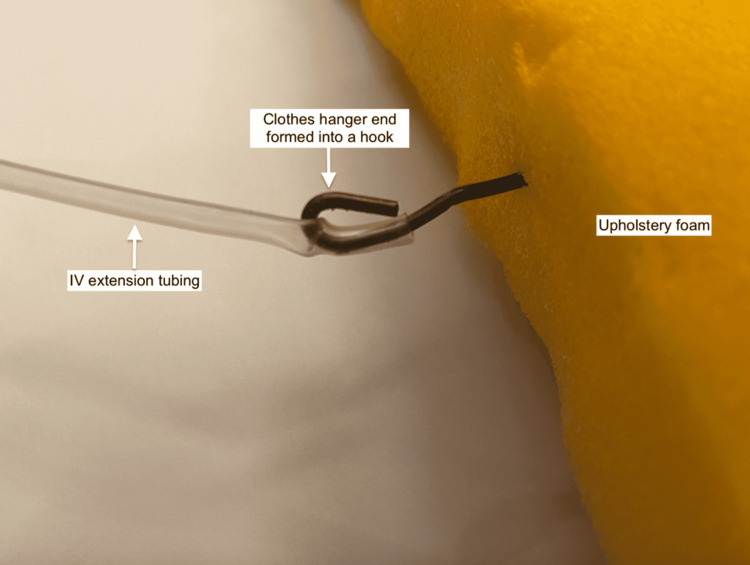
Subcutaneous bleeding apparatus

Four sets of IV extension tubing, representing blood vessels, were threaded horizontally through the foam using a coat hanger attached to the end of the tubing (Figure 8).

**Figure 9:**
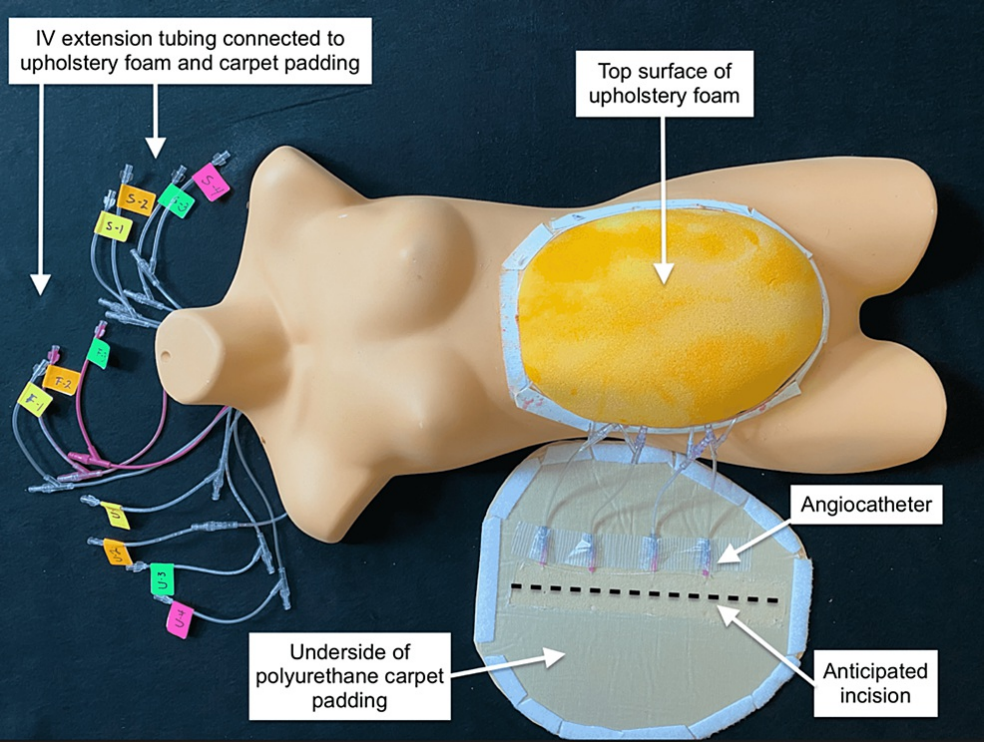
Cutaneous bleeding apparatus

The distal ends of the tubing were tied off. The foam was inserted over the uterus and tucked in under the edges of the torso’s abdominal wall, making sure the chamois was vertically oriented and faced down. The extension tubing attached to the uterine angiocatheters and the extension tubing in the upholstery foam was brought up through the neck of the plastic torso. To create the abdominal skin, a piece of ¼-in polyurethane carpet padding was shaped to cover the foam and extended to overlap the torso by an inch on all sides. Four 20-gauge X 1.88-in angiocatheters were inserted into the padding horizontally through the anticipated incision site, attached to extension tubing, and pulled through the torso’s neck as well (Figure 9).

**Figure 10:**
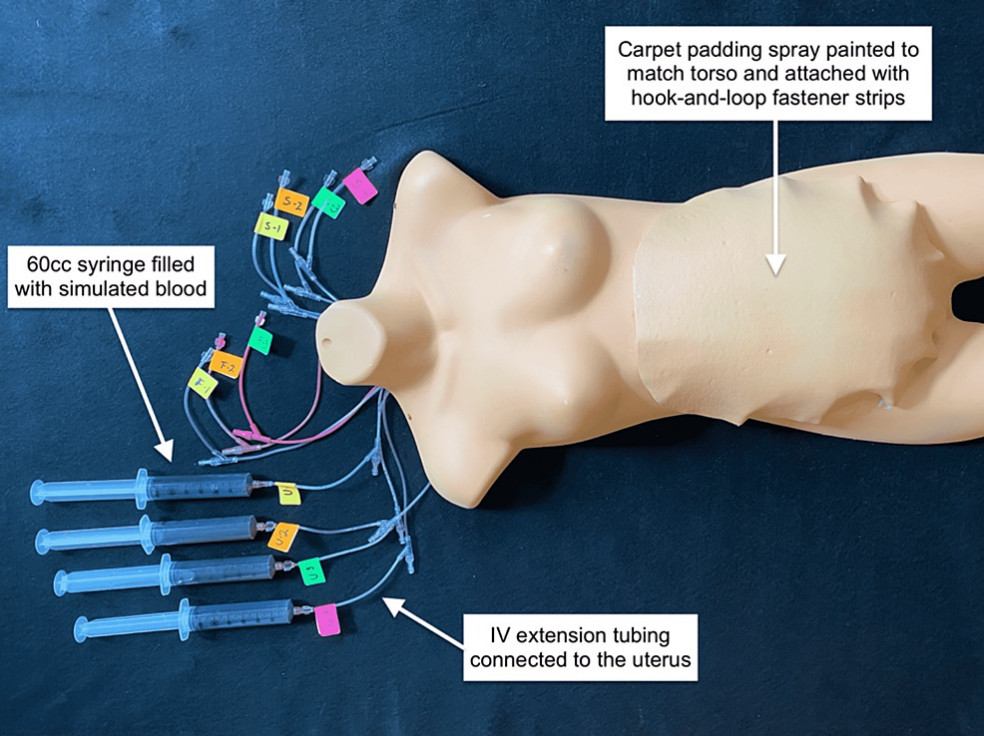
Completed table-top model (TTM)

The extension tubing coming from the uterus, subcutaneous fat, and skin were individually attached to 60-cc Luer-Lok syringes that contained simulated blood and hidden behind a barrier with a drape. The padding was attached to the torso using industrial hook and loop fastener. The torso and the abdominal skin were spray painted using a synthetic lacquer (Figure 10).

**Figure 11:**
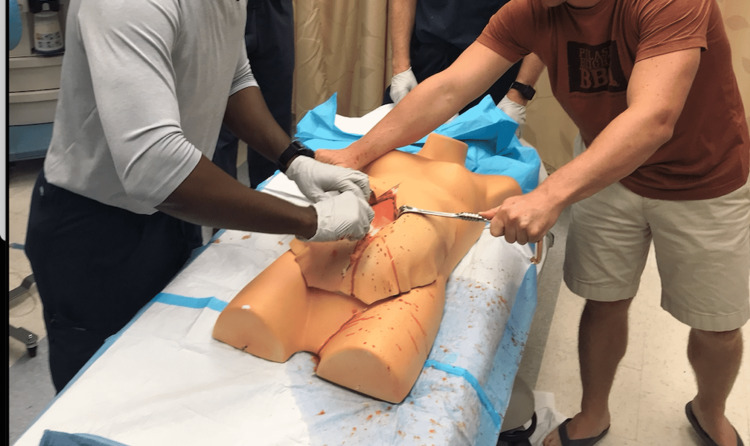
Table-top model (TTM) resuscitative hysterotomy (RH) in progress

As an incision was made through the layers of the abdominal wall, an operator at the head of the bed injected blood through the appropriate extension tubing to simulate hemorrhage (Figure 11).

Construction of the High-Fidelity Simulator Adaptated Model (HFSAM)

We were able to construct the HFSAM using mostly materials procured or purchased for the TTM. Table 2 below lists additional items that were required. The simulator was loaned to us by our simulation center.

**Table 2:** Additional items required to construct the high-fidelity simulator adaptated model (HFSAM)

Items Purchased	Quantity	Price
IV extension tubing 30 in	4	$3.80
multi-way stopcock 4 gang	2	$47.98
polyurethane upholstery foam 1 in x 2 ft x 3 ft	1	$9.99
life-size placenta model and umbilical cord	1	$55.99
Total		$117.76

**Figure 12:**
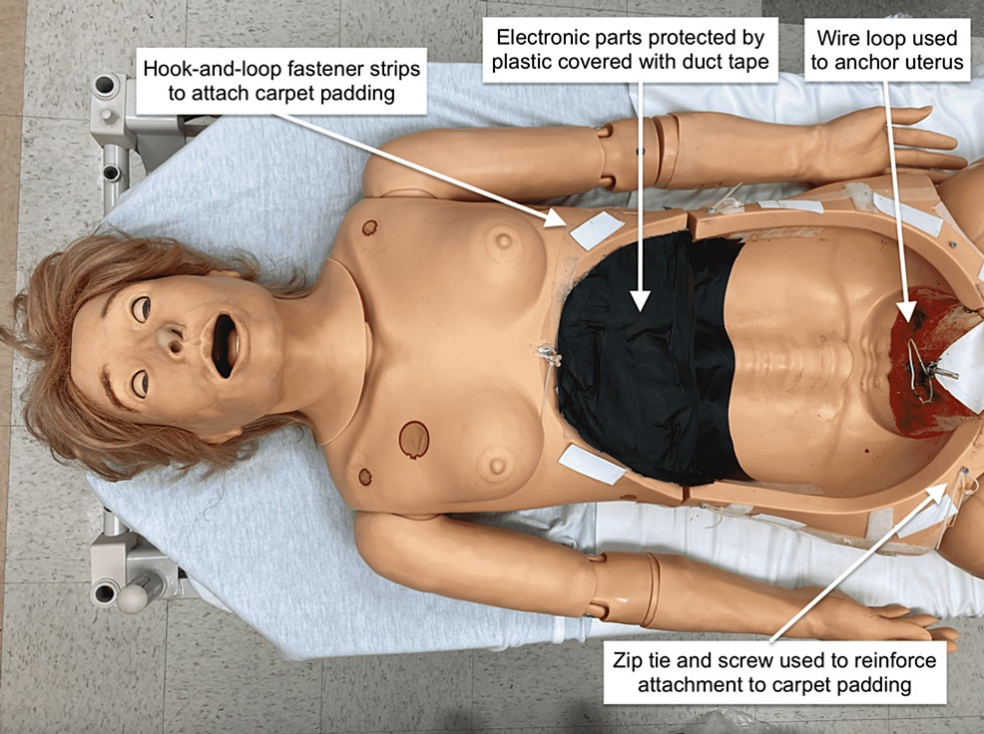
Initial modiﬁcation to the high-fidelity simulator adapted model

To adapt the TTM into a high-ﬁdelity simulator, the birthing mechanism was removed from a Gaumard Noelle® birthing simulator (Gaumard Scientific, 2012). A diaphragm was created using duct tape applied over the plastic wrap to protect electronic parts in the chest cavity. The simulator was prepared for uterine attachment by securing a wire loop attached to a zip tie to the ﬂoor of the pelvis. Hook-and-loop fastener strips were placed around the perimeter of the abdominal and pelvic cavity for skin attachment. Two screws were inserted into the perimeter of the pelvis and attached to zip tie loops which would be used to further secure the skin (Figure 12).

**Figure 13:**
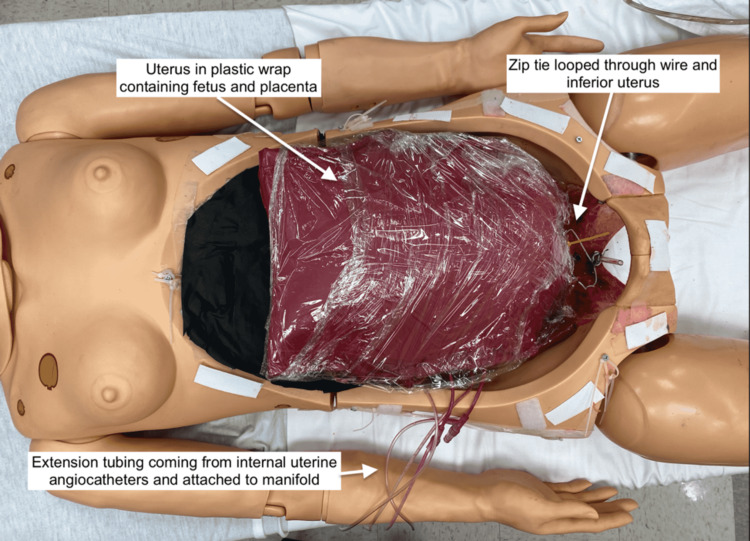
Uterus attached to simulator with peritoneal covering

The ﬂoor of the abdominal cavity was lined with plastic to contain any simulated blood and avoid staining. A uterus containing a fetus and placenta within an amniotic sac (as described above for the TTM) was covered with plastic wrap and inserted into the abdominal cavity. The inferior aspect of the uterus was anchored to the simulator using a zip tie inserted through the uterus and attached to a wire secured in the pelvic cavity (Figure 13).

**Figure 14:**
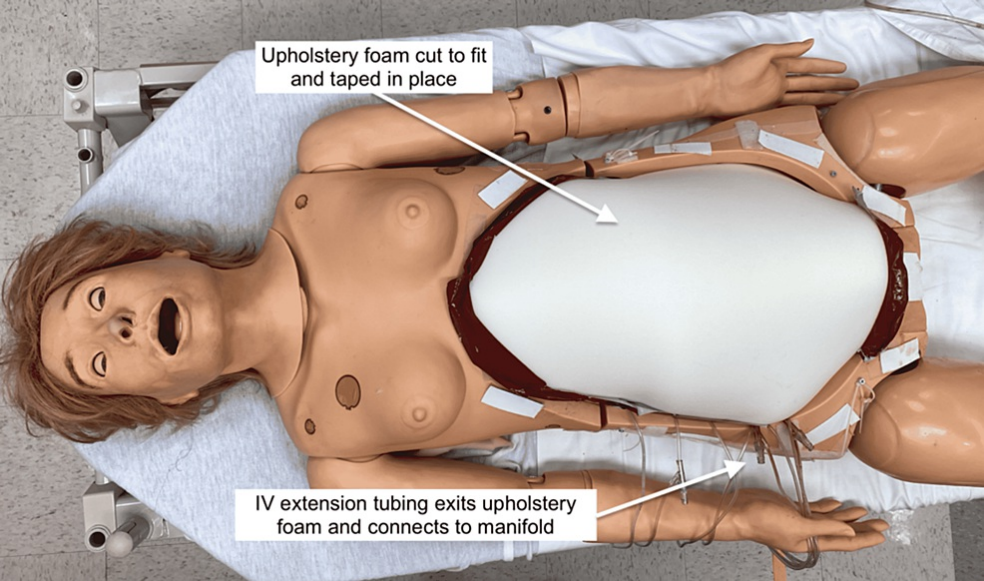
Subcutaneous layer adapted to simulator

Upholstery foam representing subcutaneous fat was cut to ﬁt the abdominal cavity. Four sets of IV extension tubing were inserted into the foam to simulate blood vessels, similar to the TTM (Figure 14).

**Figure 15:**
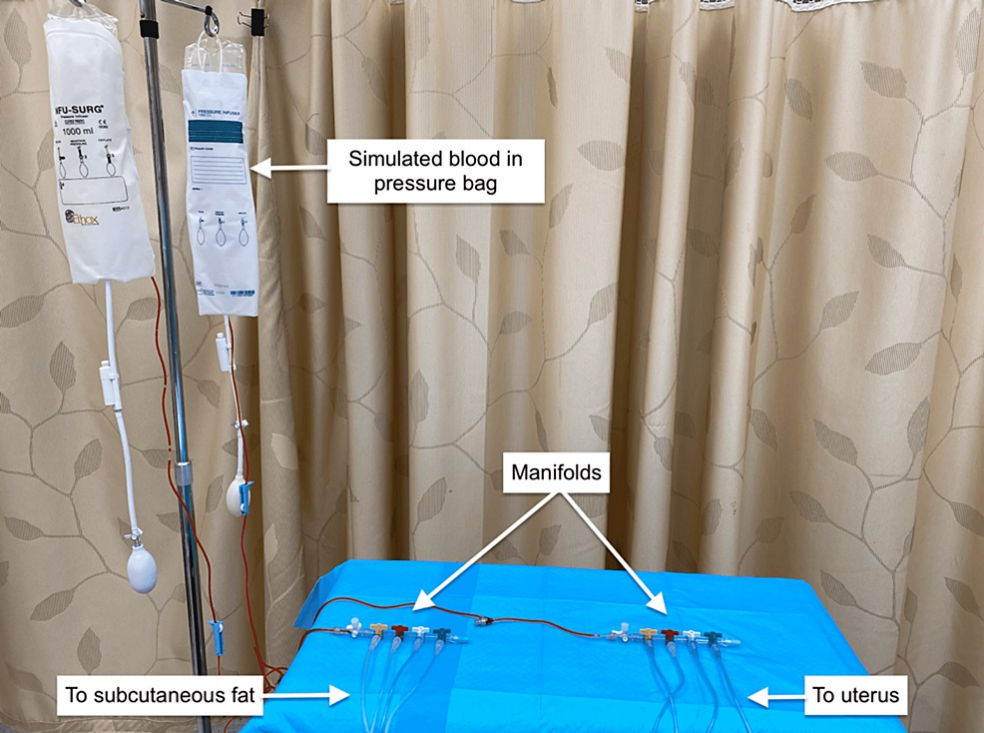
Simulator bleeding apparatus setup

The IV extension tubing from the subcutaneous fat and uterus were run out from under the abdominal wall and each attached to a stopcock manifold. Two IV bags containing simulated blood were placed inside pressure bags and connected to the manifolds (Figure 15).

**Figure 16:**
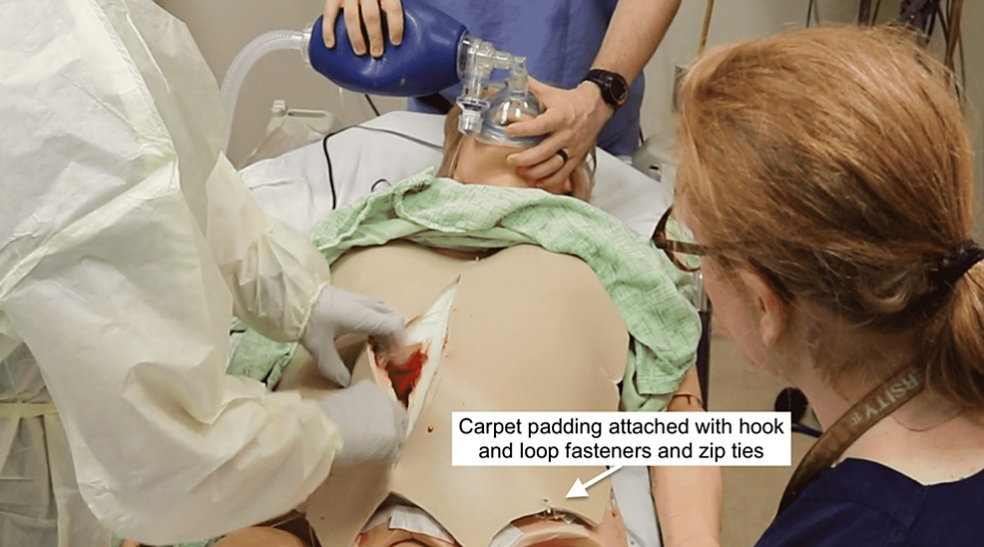
High-fidelity simulator adaptated model (HFSAM) resuscitative hysterotomy (RH) in progress

A piece of ¼-in carpet padding simulating skin was attached to the simulator using industrial-grade hook-and-loop fasteners and reinforced with zip ties inserted through the skin and attached to the simulator. As incisions were made through the abdominal wall and uterus, the appropriate stopcocks were opened to allow for simulated bleeding (Figure 16). The simulated blood used for the models was made from sweetened condensed milk, water, and food coloring.

We incorporated our models into our monthly EM residency simulation conference. The simulation scenario involved a pregnant woman in cardiac arrest and required participants to resuscitate the mother as well as perform a RH using the HFSAM. After the scenario was completed, participants performed a RH on the TTM during debriefing to serve as a hands-on demonstration to the entire learner group. Participants then completed anonymous surveys in which they rated the educational value of the session, the realism of the models, and their pre-and post-training confidence in performing a RH on a 10-point Likert scale. At the end of the quantitative survey, there was a general question regarding the overall simulation experience.

A total of 20 EM resident physicians representing all three residency classes participated in the simulation session. 11 participants (55%) served as the primary proceduralist performing a RH on either the HFSAM or the TTM, while the other nine participants assisted. All (20/20) of the participants completed the post-session survey. The residents rated the overall educational value of the training event very highly (mean 9.8 (SD 0.68)). Compared to pre-session ratings, residents reported statistically significant post-session improvements in their confidence level in performing a RH (mean 4.0 (SD 2.62) vs. mean 7.9 (SD 1.48); P<0.001). EM resident participants rated both the TTM (mean 8.9 (SD 1.15)) and HFSAM (mean 8.7 (SD 1.29)) as highly realistic and rated the bleeding as a very important aspect of the realism of the models (mean 8.6 (SD 1.74)).

## Discussion

Our goal was to develop high-fidelity training models to enhance resident confidence and proficiency in performing a RH and to prepare them for the psychological barrier inherent in performing the procedure. We sought to replicate the visual, tactile, and emotional experience of the procedure by creating models with a life-like appearance, realistic skin and soft tissue layers, and hemorrhage capability. Two models were available for each simulation session allowed over half of the residents to perform a RH. The feedback was highly positive for the overall educational experience, the realism of the models, and specifically the importance of hemorrhage occurring during the procedure. The mean resident confidence level in the ability to perform a RH doubled. Qualitative comments centered around the realism of the models and the novelty of bleeding during the RH. 

The budget required to construct our task trainers is higher than that of other published low-cost models [7-9]. However, some of the most expensive items listed in Table 1, including the plastic torso, plywood, placenta, and newborn dolls, are durable supplies that can be reused numerous times throughout multiple educational sessions. Also, when estimating cost, we assume that all items are purchased retail. The price of both models can be significantly reduced by using expired medical supplies and commonly available household products. Finally, rather than paying for a commercially constructed placenta and umbilical cord, one could make it from inexpensive materials such as padded cloth and rubber tubing. We felt that moderately higher costs for our two models was justified as it allowed us to enhance appearance as well as anatomical fidelity. 

A challenge we encountered with both models pertained to the subcutaneous bleeding apparatus. The IV extension tubing threaded through the foam padding was visible, leading some residents to avoid cutting it. This could be remedied by using tubing of smaller diameter, coloring the tubing, or using longer angiocatheters like those in the uterus. 

To our knowledge, we have developed the first non-commercial RH models that hemorrhage during incision.

## Conclusions

RH is a critically important procedure that can be life-saving for both mother and fetus. Because maternal cardiac arrest is a HALO event, developing and maintaining the skills for performing a RH is challenging. We have created two low-cost, high-fidelity RH task trainers: a stand-alone TTM and a HFSAM. Our models received very favorable feedback from our EM residents, and we hope that other training programs will find them useful as well.
